# Impact of ^18^F-FDG PET/CT on treatment of patients with differentiated thyroid carcinoma, negative ^131^I whole body scan and elevated serum thyroglobulin

**DOI:** 10.22038/AOJNMB.2021.58276.1406

**Published:** 2022

**Authors:** Raef R. Boktor, Sze Ting Lee, Salvatore U. Berlangieri, Andrew M. Scott

**Affiliations:** 1 Department of Molecular Imaging and Therapy, Austin Health, Melbourne, VIC Australia; 2 Olivia Newton-John Cancer Research Institute, Melbourne, VIC, Australia; 3 School of Cancer Medicine, La Trobe University, Melbourne, VIC, Australia; 4 Faculty of Medicine, University of Melbourne, Melbourne, VIC, Australia

**Keywords:** FDG PET, Thyroglobulin, ^ 131^I, Thyroid carcinoma

## Abstract

**Objective(s):**

^ 18^F-FDG PET/CT is increasingly performed in patients with differen-tiated thyroid cancer. The aim of this study was to assess the clinical impact of ^18^F-FDG PET/CT on the management of patients with differentiated thyroid carcinoma who had elevated serum thyroglobulin (Tg) and negative ^131^I whole body scan (WBS).

**Methods:**

67 patients with differentiated thyroid carcinoma were included in this study. The findings of ^18^F-FDG PET/CT imaging were compared with histo-pathology, follow up imaging, or clinical follow-up results. The diagnostic accuracy of ^18^F-FDG PET/CT was evaluated for the entire patient group and for those patients with stimulated serum thyroglobulin levels of less than 5, 5–10, and more than 10 pmol/L as well as for local recurrences and metastases sites. The impact of ^18^F-FDG PET/CT on therapeutic management was also evaluated.

**Results:**

30/67 patients had positive findings on ^18^F-FDG PET/CT; 28 were true-positive and 2 were false-positive. ^18^F-FDG PET/CT results were true-negative in 36 patients and false-negative in 1 patient. The overall sensitivity, specificity, accuracy, PPV and NPV of ^18^F-FDG PET/CT were, 96.5%, 94.5%, 95.5%, 93.3%, and 97.2% respectively. Positive ^18^F-FDG PET/CT findings were directly correlated with stimulated serum thyroglobulin levels, 7.1% had Tg between 5–10, and 92.9% had Tg greater than 10 pmol/L. ^18^F-FDG PET/CT had a high or moderate impact on treatment management in 28 (41.8%) of patients.

**Conclusion:**

^18^F-FDG PET/CT is able to improve diagnostic accuracy and have management impact in a therapeutically relevant way in patients with differentiated thyroid carcinoma who present with rising thyroglobulin level, negative ^131^I WBS, and clinical suspicion of recurrent disease.

## Introduction

 Serum thyroglobulin (Tg) and radioiodine whole-body scan (^131^I WBS) play important roles in the postsurgical follow-up of differentiated thyroid cancer (1–5). Elevated Tg level is a sensitive marker for residual or recurrent disease (6). Elevated serum Tg 

concentration is usually associated with positive ^131^I WBS (3, 7). Studies have shown that approxi-mately three quarters of recurrences and metastases from well-differentiated thyroid carcinoma are capable of concentrating ^131^I (8). This means that a quarter of patients with recurrent or metastatic disease will present

with an elevated Tg level and negative ^131^I WBS. This constitutes a therapeutic dilemma, because these patients have elevated Tg levels but no identifiable focus of disease on a diagnostic ^131^I scan.

 There are two principal factors accounting for the discrepancy between serum Tg levels and ^131^I WBS. Firstly, the tumour volume might be too small to be detected by ^131^I WBS, and secondly, tumour cells may lose the ability to trap radioiodine while still retaining the ability to secrete Tg (9). It is important to localize tumour sites to direct appropriate treatment, such as surgery or external-beam radiotherapy (10, 11). 

 Morphologic imaging modalities such as sonography, CT, and MRI may be effective in localizing such lesions especially in the case of local recurrences or in the evaluation of suggestive remote sites, but these modalities are not practical for whole-body evaluation (4). Furthermore, they may not be able to distinguish active disease from the fibrotic residue of previously treated disease. Some SPECT radiopharmaceuticals, such as 201Tl, ^99m^Tc-sestamibi, and ^111^In-octreotide, have demons-trated some usefulness in the identification of disease sites when ^131^1 fails and Tg levels are elevated (12-15). With all these approaches, positive findings are useful but negative resultsare less than fully reassuring when Tg is elevated.

 Malignant cells frequently exhibit increased glucose metabolism that can be visualized on ^18^F-FDG PET. Differentiated thyroid cancer is not generally characterized by a marked increased ^18^F-FDG uptake (16). Some groups have studied ^18^F-FDG PET in detecting metastatic or recurrent non iodine-avid lesions (6, 10, 17-20, 28-31). The purpose of this study was to assess the clinical impact of contemporary ^18^F-FDG PET/CT on the management of patients with differentiated thyroid carcinoma who had elevated Tg levels and negative ^131^I WBS.

## Methods

 A total of 117 patients with thyroid carcinoma underwent 176 ^18^F-FDG PET/CT scans at our institution. There were 96 patients with differentiated thyroid carcinoma, 16 patients with medullary thyroid cancer, 3 patients with lymphoma and 2 patients with anaplastic thyroid carcinoma. Our analysis included only the 67 patients with pathologically proven differen-tiated thyroid carcinoma and negative ^131^I WBS (Table 1). This study was approved by the Institutional Review Board of the Austin Hospital.

**Table 1:** Summary of Patient Characteristics

**Histology**	N*	**%**
Follicular Thyroid Cancer	4	6
Papillary Thyroid Cancer	57	85
Hurthle cell Carcinoma	3	4.5
Insular Carcinoma	1	1.5
Poorly Differentiated Thyroid cancer	2	3

*No. of patients (Total n= 67). Male: 17. Female: 50

 All the patients had elevated stimulated Tg level (≥1 pmol/L or equivalent in ng/ml) in absence of high level anti-Tg antibodies. The findings of ^18^F-FDG PET/CT and ^131^I WBS imaging were compared with histopathology, follow-up imaging, or clinical follow-up (including Tg level) results as gold standards. The results were used for further clinical decision making. All the patients included in this study were initially treated by total thyroidectomy followed by ^131^I ablation. The ablation doses ranged from 2.75 to 5.5 GBq (75–150 mCi) of ^131^I.

 Sixty seven patients presented for ^18^F-FDG PET/CT imaging because of elevated Tg level and negative ^131^I WBS. Stimulated Tg level at the time of ^18^F-FDG PET/CT was as follows: 44 patients had Tg level >10 pmol/L (range 12-56,500 pmol/L), 10 patients had Tg level between 5-10 pmol/L, and 13 patients had Tg level < 5 pmol/L. All patient Tg autoantibodies 

levels were <10 kunits/L. All the Tg assays were performed in the same Institutional laboratory.

^131^ ***I WBS scan***

^131^I WBS were obtained using a dual head Philips Skylight SPECT gamma camera (Philips Medical System, Cleveland, Ohio, USA) with a high energy parallel hole collimator. Anterior and posterior whole body images were acquired at speed of 8 cm/min. Additional 5 minutes planar images of the neck and chest were acquired with the neck in extended position. SPECT was not routinely performed.

^18^ ***F-FDG PET/CT imaging***

^18^F-FDG PET/CT was performed 1-2 week after ^131^I WBS. Patients fasted for at least 6 hours before the scan. An intravenous cannula was placed for radiopharmaceutical administration, and the blood glucose level was measured before tracer injection, and ranged between 3.5-6.2 mmol/L at the time of ^18^F-FDG injection. Each patient received 370–460 MBq (10–12.5 mCi) of ^18^F-FDG intravenously. After tracer injection, the patients rested in lead lined semi-dim room during the ^18^F-FDG uptake period. PET/CT acquisition started 60 min after injection of the ^18^F-FDG using a Gemini PET/CT scanner (Philips Medical System, Ohio, USA). Whole-body scans were acquired from mid thighs to the base of skull. Low dose CT was performed for attenuation correction and anatomical correla-tion. The scanning parameters for whole-body CT craniocaudal scanning were 140 kV, 30 mAs (may be increased according to patient body mass index), 5-mm collimation, and a pitch of 1.5. 

 The helical CT scan was reconstructed by iterative method into 512×512 pixel images with a slice thickness of 6.5 mm.

^18^ ***F-FDG PET/CT analysis***

 All ^18^F-FDG PET/CT scans were interpreted by experienced nuclear medicine physicians. The leading criterion for ^18^F-FDG PET/CT interpretation was the presence of focally increased FDG uptake. Therefore, any increased FDG uptake was compared with anatomic findings on CT. All areas with abnormally increased FDG uptake corresponding to a CT abnormality (tissue mass or lymph node) were interpreted as positive for recurrent or metastatic disease. CT findings which did not correspond to abnormal FDG uptake were reported as non-FDG avid lesions. The results of the ^18^F-FDG PET/CT scan was correlated with other investigations, which included the results from subsequent imaging modalities such as neck ultrasound, CT, and post-radioiodine ablation scan; Tg level; histologic examination of surgical specimens as well as clinical follow up. The ^18^F-FDG PET/CT findings were classified as follows:

- Lesions were true-positive if positive findings on ^18^F-FDG PET/CT were confirmed by histologic examination or were confirmed by other imaging modalities in the presence of persistent abnormal or increasing Tg, or decreasing Tg after active therapy.
- Lesions were false-positive if biopsy samples of suspicious lesions were negative or the lesions had resolved on subsequent follow-up imaging or reduced Tg without active therapy.
- Lesions were true-negative if elevated Tg had normalized without treatment, and if metastatic disease was not evident on subsequent follow-up. Follow-up was continued in all patients with true-negative lesions for 12-48 months (mean 22 months)
- Lesions were false-negative if the findings of ^18^F-FDG PET/CT were negative and metastatic thyroid cancer was found on histologic examination, or if disease progression was seen on other imaging modalities, or rising Tg levels.

Same definitions and classifications of true positive, true negative, and false positive and false negative were applied on ^131^I WBS.

 The impact of ^18^F-FDG PET/CT on management changes were determined according to the following definitions (21):

- No impact: ^18^F-FDG PET/CT result was consistent with planned management, and treatment modality, or intent was unchanged.
- Low impact: ^18^F-FDG PET/CT result indicated that a different planned manage-ment may have been appropriate, but management was not changed by the ^18^F-FDG PET/CT result.
- Moderate impact: Treatment modality/ intent unchanged, but the planned procedure, dose or mode of delivery was altered by ^18^F-FDG PET/CT result
- High impact: Treatment modality/intent was changed by ^18^F-FDG PET/CT result.

***Statistical Analysis***

 Sensitivity, specificity, positive and negative predictive values, and accuracy were calculated for ^18^F-FDG PET/CT. Correlating PET/CT findings with Tg levels <5, 5–10, or >10 pmol/L was also performed.

## Results

 There were 67 patients (17 male, 50 female; age range, 21–87 years) with pathologically proven differentiated thyroid carcinoma and negative ^131^I WBS included in this study (Table 1). A total of 36/67 patients were true negative confirmed by decreasing Tg levels, negative ^18^F-FDG PET/CT scans and no clinical evidence of disease over the follow up period up to 48 months. ^18^F-FDG PET/CT findings were positive in 30/67 (44.8%) patients with negative ^131^I WBS; true positive in 28 (41.8%) patients; and false positive in 2 patients. One patient was false negative on both ^18^F-FDG PET/CT and ^131^I WBS. Of the 28 patients with true positive ^18^F-FDG PET/CT scans but negative ^131^I WBS, 17 patients had local and cervical nodal recurrence, 6 patients had pulmonary metastases, 1 patient had mediastinal nodal metastases and 4 patients had bone metastases. The Tg levels in these patients were >10 pmol/L in 26 patients and between 5-10 pmol/L in 2 patients with cervical nodal recurrence.

 There were 9 out of the 28 true positive patients who showed nodal disease for which surgery was performed and results were confirmed by histopathology. A total of 11 patients were treated with ^131^I for local non-resectable recurrence, pulmonary and/or bone metastases with positive post-therapy iodine scan and/or decreasing Tg after therapy. There were 8 patients referred to the oncology and radiotherapy units based on multiple FDG-avid lesions, elevated Tg and negative ^131^I WBS scan, and were treated accordingly with decreasing level of Tg after therapy.

 The findings of ^18^F-FDG PET/CT were false positive in 2 patients. One patient was suspected to have bone marrow infiltration on ^18^F-FDG PET/CT given the elevated Tg level (87 pmol/L) and this proved to be a false positive by negative bone marrow biopsy and decreasing Tg on follow-up with no active treatment. One patient with anterior mediastinal uptake on ^18^F-FDG PET/CT and elevated Tg (4 pmol/L) showed decreasing Tg on follow-up over 4 years with no active treatment or clinical evidence of disease progression.

 The findings of ^18^F-FDG PET/CT were true-negative in 36/67 (53.7%) patients as confirmed by decreasing Tg levels and negative follow-up ^131^I WBS and/or PET/CT scans without clinical evidence of disease over the follow-up period (range 12-48 months, mean 22 months). A total of 12 of these 36 patients with negative ^18^F-FDG PET/CT scans had Tg <5 pmol/L, 8 patients had Tg between 5-10 pmol/L and 16 patients had Tg >10 pmol/L (Table 2).

 The findings of ^18^F-FDG PET/CT were false-negative in 1 patient who had Tg >10 pmol/L. This patient subsequently showed local recurrence and cervical nodal metastases 12 months later on neck ultrasound and CT.

 The sensitivity of ^18^F-FDG PET/CT in detecting differentiated thyroid cancer metastasis or recurrence was 96.5%, specificity 94.5%, accuracy 95.5%, positive predictive value (PPV) 93.3% and negative predictive value (NPV) 97.2%.

***Diagnostic Accuracy of*** ^18^ ***F-FDG PET/CT and Serum Tg Levels***

 The findings of ^18^F-FDG PET/CT were true positive in 2 (7.1%) patients with Tg between 5-10 pmol/L, and 26 (92.9%) patients had Tg levels >10 pmol/L. The findings of ^18^F-FDG PET/CT were true negative in 12 (33.3%) patients with Tg <5 pmol/L, 8 (22.2%) patients with Tg 5-10 pmol/L and 16 (44.5%) patients with Tg level >10 pmol/L (Table 2).

**Table 2:** ^18^F-FDG PET/CT Findings Compared with Serum Tg levels

	^18^F-FDG PET/CT			
	True Positive (n=28)	True Negative (n=36)	False Positive (n=2)	False Negative (n=1)
Tg: < 5 pmol/L	n=0 (0%)	n=12(33.3%)	1	0
Tg: 5-10 pmol/L	n=2 (7.1%)	n=8 (22.2%)	0	0
Tg: >10 pmol/L	n=26 (92.9%)	n=16 (44.5%)	1	1

***Stratification of*** ^18^ ***F-FDG PET/CT &*** ^131^ ***I WBS according to pathological data***

 The 31 false negative ^131^I cases were 22 papillary, 4 follicular, 3 Hurthle cell, 1 poorly differentiated and 1 insular. There were 17/31 patients who had lymph nodal involvement initially and 4 had lymphatic and/or vascular invasion. Of the 28 true positive ^18^F-FDG PET/CT cases, 19 were papillary, 4 were follicular, 3 were Hurthle cell, 1 was poorly differentiated and 1 was insular. There were 22 patients who had lymph nodal disease initially and 5 had lymphatic and/or vascular invasion. 5 were stage II, 19 were stage III and 4 were stage IV.

***Management change after*** ^18^ ***F-FDG PET/CT***

**Figure 1:**
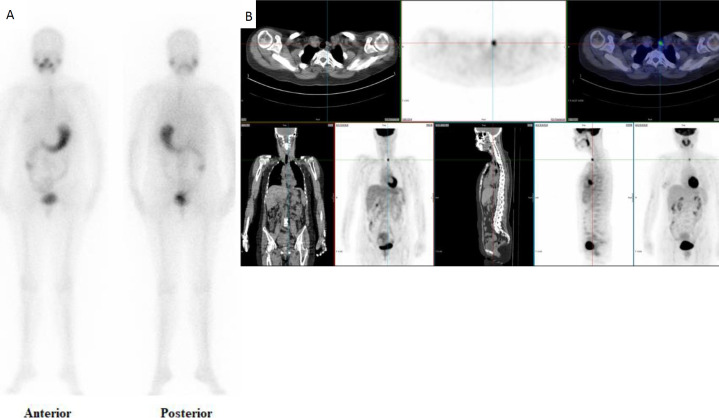
A 44 year old woman who had undergone total thyroidectomy and ablative radioiodine treatment, presented after two years with elevated Tg level (106 pmol/L) and negative ^131^I WBS (**A**). ^18^F-FDG PET/CT showed small focal intense FDG uptake in left cervical lymph node (**B**). The patient was treated with ^131^I ablation with subsequent decrease in Tg level and negative follow up ^18^F-FDG PET/CT

 In 19 (28.4%) patients, ^18^F-FDG PET/CT had a high impact on patient management. A total of 9 patients had surgery based on the ^18^F-FDG PET/CT findings of positive cervical nodes. Ultrasound results in all the 9 patients were either negative or inconclusive. There were 8 patients referred to oncology and radiotherapy for further treatment based on multiple FDG-avid lesions, high Tg level and negative ^131^I WBS. A further 2 patients proceeded to ^131^I therapy (Figure 1). In this group ^18^F-FDG PET/CT has changed the modality of treatment.

 In 9/67 (13.4%) patients, ^18^F-FDG PET/CT has a moderate impact on patient management. In 1 patient out of the 9 there were mediastinal nodes seen on ^18^F-FDG PET/CT. There were 4 patients with pulmonary metastases and 4 patients had local and distant recurrence on ^18^F-FDG PET/CT with negative ^131^I scans. All patients in this group had a Tg level >10 pmol/L. The primary plan in these patients was to give ^131^I therapy in the context of an elevated Tg. The dose of ^131^I was changed in view of the ^18^F-FDG PET/CT result.

 In 36 (53.7%) patients who had negative ^18^F-FDG PET/CT and ^131^I scan, ^18^F-FDG PET/CT had low management impact and the patients were clinically followed up with Tg depending on the result of PET/CT. The main value of ^18^F-FDG PET/CT in this group was assurance of the planned line of treatment and avoiding further unnecessary investigation and/or treatment that may be decided based on the elevated Tg level.

 In 3 (4.5%) patients, ^18^F-FDG PET/CT had no impact on patient management, 1 was false negative on ^18^F-FDG PET/CT and ^131^I WBS, and 2 false positive on PET/CT.

## Discussion

 After the ablation of residual thyroid tissue, measurement of serum Tg levels and ^131^I imaging usually provide sensitive tools for the early detection of recurrence. Unfortunately, some patients with elevated Tg had tumours that did not concentrate ^131^I even after administration of therapeutic doses, because of an impaired ability to trap iodine. ^18^F-FDG PET offers an accurate method of detecting such tumours. The sensitivity of ^18^F-FDG PET is determined by the metabolic activity of recurrent or metastatic thyroid cancer. An inverse relationship has been reported between the ability to concentrate radioiodine and the uptake of ^18^F-FDG in thyroid cancer (7, 16, 19, 31). In most tumours, ^18^F-FDG uptake increases with the level of dedifferentiation (16) and has been found to correlate with the expression of the glucose transporter protein-1 (22). ^18^F-FDG PET is especially helpful for detecting recurrent or metastatic lesions in patients with increased Tg levels and negative radioiodine WBS findings (9-11, 16-20, 29, 32, 33).

 The previously published sensitivity and specificity of ^18^F-FDG in different types of differentiated thyroid carcinoma ranges from 71% to 94% and from 73% to 95% respectively, with a pooled sensitivity of 88.5% and pooled specificity of 84.7% (23). A recently published meta-analysis reported a pooled sensitivity of 86% and specificity of 84% (29). In one study of 64 patients with suspected recurrent disease, the sensitivity of ^18^F-FDG PET/CT was reported to be 69.4% (11). In our study of 67 patients, we found our results are comparable with the most of the published results apart from a higher negative predictive value, with the overall PET/CT sensitivity and specificity, accuracy, PPV, and NPV of 96.5%, 94.5%, 95.5%, 93.3% and 97.2% respectively, for detecting recurrent and/or metastatic thyroid cancer. 

 A dual centre study performed by Lee et al in 286 patients showed that ^18^F-FDG PET/CT was able to identify additional 39 (14%) positive case that were not identified on post therapy ^131^I WBS. They reported that the mean serum Tg levels in patients with positive and negative ^18^F-FDG PET/CT lesions were 121.0±203.1 ng/mL (range=0.4–1000.0 ng/mL) and 7.8±19.0 ng/mL (range=0.1–167.7 ng/mL), respectively. There was a statistically significant difference in the serum Tg levels between the 2 groups (P=0.0001) (28). Shammas et al carried out a study on 61 patients with differentiated thyroid carcinoma and reported PET/CT sensitivity, specificity, accuracy, PPV & NPV of 68.4%, 82.4%, 73.8%, 86.7%, 61.3% respectively. They stated that the sensitivities of ^18^F-FDG PET/CT increases with the increase in serum Tg levels, with reported sensitivities of 60%, 63% & 72% for Tg levels <5, between 5–10, and >10 ng/mL respectively(17). 

 In our study, 67 patients presented with negative ^131^I WBS and elevated Tg level, ^18^F-FDG PET detected disease in 38.8%, 7.3 % & 0% for Tg level >10 pmol/L, 5-10 pmol/L and <5 pmol/L respectively. This percentage is different from what was reported by Shammas et al. We attributed this to the difference in using two different Tg measurement units (ng/ml and pmol/L, conversion factor ng/ml x 2.25= pmol/L). However, it is clear from the results of both studies that the ability of PET to detect recurrence/metastasis is directly related to the level of increase in serum Tg.

 Detection of local recurrence or cervical nodal metastases is difficult by various imaging modalities. CT neck is difficult to interpret without administering intravenous contrast which is the case in thyroid carcinoma patient in order not to block the ^131^I uptake. MRI is highly sensitive however it lacks specificity for disease detection (4, 25). Ultrasound has an important role in restaging patients, with advances in technology such as high-resolution transducers, color flow Doppler, and power Doppler providing detailed information and improved detection of local recurrences and lymph node metastases (26). While all the patients included in this analysis has negative ^131^I-WBS, SPECT/CT was not routinely performed which may have revealed low grade uptake in some lesions, however the yield of SPECT/CT positive results when ^131^I-WBS is negative may be quite low (34). 

^18^F-FDG PET/CT has a superior advantage of whole body imaging to detect distant metastases in patients with negative whole body radioiodine scan and rising Tg levels. 

 An advantage of ^18^F-FDG PET/CT is the ability to identify thyroid cancer recurrences and metastases in soft tissue, lymph nodes, liver, lungs, and bone in a single imaging procedure. In a meta-analysis study performed by Dong et al, of 237 suspicious recurrent and metastatic lesions from six studies, they found ^18^F-FDG PET to be beneficial in the diagnosis of recurrent and metastatic lesions, with a sensitivity and specificity of 91.6% and 77.5%, respectively (23).

 It is well known that^ 18^F-FDG PET is not able to adequately assess miliary lung metastases smaller than 6 mm. This may be related to motion artifacts on respiration or from a lower metabolic activity of the lung metastases (16). In our study, ^18^F-FDG PET/CT identified 11 (16.4%) patients with mediastinal, lung and bone metastases. A total of 6 of the 11 patients had pulmonary metastases and were detected by ^18^F-FDG PET, all of them with pulmonary nodules ≥10 mm.

^18^F-FDG PET/CT had a management impact in 64/67 (95.5%) patients included in this study, and of these moderate or high impact was seen in 28/67 (41.8%) of patients. A crucial advantage of ^18^F-FDG PET/CT is the precise detection and localization of local recurrences and/or distant metastatic disease, assisting with choosing the appropriate line of treatment. This information, for example, properly adjusted the dose of^ 131^I therapy, excluded patients from further iodine therapy, improved surgical planning and target definition for external beam radiation. ^18^F-FDG PET/CT directed the treatment to neck dissections, higher doses of ^131^I therapy, radiation therapy, or chemotherapy in cases with multiple non-iodine avid metastases.

 Although the exact contribution of ^18^F-FDG PET/CT to patient management may be difficult to quantify, we subdivided its impact into low, moderate and high according to previously published criteria for management change (21). We found our overall management impact (high and moderate) results similar to what have been previously stated in the literature (41.8%), and confirmation of a negative result (53.7% of our patients) was categorised as low impact. In one study, management change was found in 29/37 (78%) patients (4), and in another there were 19/40 (48%) patients where ^18^F-FDG changed management (24). 

 Wang et al suggested that ^18^F-FDG uptake may correlate with the TNM stage of thyroid cancer, but that it is not as effective in low stage differentiated thyroid carcinoma as in high stage patients (27). Our results are similar, as we found that 23/28 (82.1%) true positive ^18^F-FDG PET/CT patients were stage III &IV in the primary histopathology. We also found that 22/28 (78.5%) of these patients had positive lymph node metastases initially.

## Conclusion

^18^F-FDG PET/CT is able to improve diagnostic accuracy and impacts on patient management in patients with differentiated thyroid carcinoma who present with rising Tg level and negative ^131^I WBS.

 No disclosures or grants involved in this research.
